# Reflections on Palliative Care from the Jewish and Islamic Tradition

**DOI:** 10.1155/2012/693092

**Published:** 2011-12-01

**Authors:** Michael Schultz, Kassim Baddarni, Gil Bar-Sela

**Affiliations:** ^1^Division of Oncology, Rambam Health Care Campus, Faculty of Medicine, Technion Israel Institute of Technology, P.O. Box 9602, Haifa 31096, Israel; ^2^Al-Taj for Health and Heritage Organization, Arraba 30812, Israel

## Abstract

Spiritual care is a vital part of holistic patient care. Awareness of common patient beliefs will facilitate discussions about spirituality. Such conversations are inherently good for the patient, deepen the caring staff-patient-family relationship, and enhance understanding of how beliefs influence care decisions. All healthcare providers are likely to encounter Muslim patients, yet many lack basic knowledge of the Muslim faith and of the applications of Islamic teachings to palliative care. Similarly, some of the concepts underlying positive Jewish approaches to palliative care are not well known. We outline Jewish and Islamic attitudes toward suffering, treatment, and the end of life. We discuss our religions' approaches to treatments deemed unnecessary by medical staff, and consider some of the cultural reasons that patients and family members might object to palliative care, concluding with specific suggestions for the medical team.

## 1. Introduction

Holistic patient care must relate to the spiritual aspect of patients' experience and concerns. This is especially clear in an area such as the Middle East, where religious beliefs are strong and widespread, but holds true anywhere, since spirituality is a universal part of the human experience [1]. A truly integrative, holistic approach is one that addresses all aspects of the patient experience—the biopsychosocial-spiritual model [2, 3]. Religion and spirituality are important for a majority of our patients, yet their spiritual needs are not supported by the medical team most of the time [4]. The integration of spiritual care into the team approach can pay significant dividends, as spiritual care in particular and spiritual wellbeing in general are correlated with higher patient quality of life [4, 5], reduced anxiety [6, 7], reduced end-of-life despair and depression [8, 9], and shorter hospital stays [6, 7]. Even and especially at the end of life, the spiritual dimension is a very significant part of the lived experience: “As physical health wanes, spiritual health may increasingly play a central role in determining patient well-being” [4]. Religious struggle, on the other hand, can have a negative impact on well-being or even on mortality [10]. Faith is also significant for medical decision making [4, 11].

What role can the medical team play in addressing patient spirituality? Aside from the focused spiritual care provided by the chaplains, all team members have a crucial role to play in engaging patients around this part of their experience of illness. By asking patients directly about their spirituality and religious beliefs, staff better understand patients as a whole person, deepen the caring relationship, build trust, and can potentially uncover spiritual distress or spiritual beliefs that will impact on decision making [12, 13]. McCord et al. found that a large majority of patients wanted staff to discuss spiritual matters with them, and the primary reason for that desire was in order to increase patient-staff understanding [14]. Yet many doctors and nurses feel hesitant to bring up spirituality with patients. One of the factors leading to this reluctance may be unfamiliarity with specific religions' beliefs relating to illness, treatment, and death.

In Israel, the two predominant religions are Judaism and Islam, both of which are well represented in the rest of the world as well. Over 19 million Muslims have made their homes in the West. These communities are heterogeneous in many aspects—in terms of dress, diet, language, and ethnic origin [15]. Consequently, more and more doctors and nurses will come across Muslim patients in the course of their work. Building on the existing literature [15–24], this review of the Muslim approach to illness and death and the beliefs and needs of the Muslim patient will open the door to fruitful communication between Muslim patients and their health care professionals with respect to their spiritual beliefs and needs. The outcome will ensure improvement of care and mutual respect while preventing embarrassment and confrontation. Cultural competence of Muslim spiritual and religious beliefs constitutes a critical component of total care. Though there is of course a range of beliefs and religiosity in Islam, as in all religions, strong adherence to the approaches and beliefs outlined here is widespread in our country.

Judaism, perhaps more so than Islam, has a wide range of perspectives on almost every issue. Rather than focusing on legal issues, as other recent excellent works have done [25–27], we will discuss some of the range of beliefs in Judaism regarding questions of illness, treatment, and the end of life. In so doing, we hope to provide the medical practitioner with a deeper understanding of the religious thinking that may underlie a patient or family member's response to the medical situation, and ease that practitioner's entry into a conversation about spirituality with the patient or their family, thereby deepening the caring relationship and better understanding the patient as a whole person. Additionally, and perhaps most importantly, we see in our work on a daily basis that just speaking about spirituality is a way for patients to reconnect to and “touch” their own spirituality, and that alone provides a great deal of comfort and strength.

It may even be that in specific cases of spiritual struggle, where a patient or family member is having a hard time reconciling their desire for a more palliative approach to care with their self-perception that Judaism does not allow them to do so, that the perspectives offered in this article will help enable staff to share the perspective with them that such a contradiction might not exist. In general, the principles of palliative care are largely in accord with Jewish, as well as Islamic, tradition [26]. In Israel in particular, the power of traditional beliefs is strong for a considerable majority of the population, many more than those who preserve traditional practices, and of course belief only strengthens in the face of serious illness.

We will draw on our experience in the field of Israeli professional spiritual care, a young field that has developed in the last decade and ongoing research has documented its progress [28, 29].

In this discussion, our goal is not to consider the religious dimensions of end-of-life questions, such as disconnection from a ventilator. Rather, our focus will be on examining Judaism's and Islam's attitudes toward illness and the end of life, since these are essential to considering a palliative approach. In that vein, we will consider cultural concerns that arise around end-of-life care, and will examine some approaches to thinking about treatment decisions when medical hope for a cure greatly diminishes.

## 2. Attitudes toward Suffering and Treatment

One of the crucial foundations of the palliative approach is the understanding that treatment of serious illness is not only about the tension between life and death, but also includes reducing suffering and improving the patient's quality of life. In Judaism, the Biblical commandment “and you shall return it (a lost object) to him (its owner)” (Deuteronomy 22 : 4) is the source for the doctors' obligation to heal their patients. If a doctor has it in his power to “return” to the patient that which he has lost, namely, his health, then he must do so. However, there is no reason why this need be limited to his health only. As the medieval scholar Maimonides writes, this commandment broadly obtains to returning “his body, his money, and his mental health.” (translations are the authors' [30]). In this vein, the obligation to provide healing includes returning to a patient his lost quality of life and returning to him the previous form of life that he had enjoyed—life that was not full of suffering.

In Islamic belief, suffering plays an important role in life. For the Muslim, sickness and suffering are a part of life: a matter of coincidence, an attack of the evil eye, or a spiritual test from the creator. Emotional and physical suffering caused by illness is regarded as a test of faith in God, expunging the sins of the Muslim [31]. Sickness should wake people up from heedlessness, guide them to give up their sins, make them think about the hereafter, and lead them to pious foundations. It should make people more thankful to Allah and teach them the necessity of taking better care of their health and making better use of their life—something they may not have realized before. Illness should teach them to understand other sick and pained people better, to feel sorry for them, and to help them. Going through suffering also raises their ranks and degrees higher in the hereafter. According to the Islamic philosophy of life, there is a transcendental dimension to pain and suffering. Pain is a form of test or trial, to confirm a believer's spiritual station [32]. Suffering is considered a part of life, and forbearance of hardship is greatly rewarded in Islam. In particular, forbearance of an illness leads to expiation of sins in Islam [33]. The Quran tells us that those who claim to believe in Allah will not be left alone after a proclamation of their belief and asserts that believers will be put to the test in various ways: “Be sure that we shall test you with something of fear and hunger, some loss in goods or lives or the fruits of your toil, but give glad tidings to those who patiently preserve” [34] (2 : 155). Islam teaches that pain and suffering delete sins: “And bear in patience whatever (ill) may befall you: this, behold, is something to set one's heart upon” [34] (31 : 17). The Prophet (peace be upon him) said that “When the believer is afflicted with pain, even that of a prick of a thorn or more, God forgives his sins, and his wrongdoings are discarded as a tree sheds off its leaves.”

At the same time, treatment to reduce pain and suffering is mandated in Islam. The Islamic teaching encourages Muslims to seek treatment when they fall sick [31], “Seek treatment, because Allah did not send down a sickness but has sent down a medication for it, except for death.” The majority of traditional scholars viewed medical treatment as permissible in cases of chronic illness and an obligation in cases of emergency in which loss of life would occur if the person was not treated [15]. Pain relief by analgesic, including morphine, to prevent suffering is allowed and recommended, even if it hastens death, since actions are judged by their intention. The Muslim believes that pain expunges sins, but pain must be treated because God opposes human suffering; (see Jotkowitz and Zivotofsky [25] for an extended discussion of the varying, though similar, Jewish approaches to pain relief).

## 3. Thinking about Death

A second foundational aspect of the palliative approach is that death is not the enemy. In Islam, death is inevitable and occurs only with a command from God: “Every soul shall have a taste of death: in the end to Us shall you be brought back” [34] (29 : 57). It also states, “Wherever you are, death will find you out, even if you are in towers built up strong and high” [34] (4 : 78). “From it (the earth) did We create you, and into it shall We return you, and from it shall We make you appear once again” [34] (20 : 55), referring to life after death. Death should not be resisted or fought against, but rather it is something to be accepted as part of the overall divine plan [35]. “It is God who creates you and takes your souls at death” [34] (16 : 70). When death is approaching, believers should pronounce the Faith of Testimony (*Shahada*): “There is no god but Allah, and Muhammad is the messenger of Allah.” This short, important ritual consolidates the dying person's expectation that death is not the end, and that he or she is now entering the world of the divine with the proper attitude. Death is the will of God: “It is not possible for a soul to die except with the permission of God at a term set down on record” [34] (3 : 139). The only guarantee that comes along with birth is death. “To God we belong and to Him is our return” [34] (2 : 156). Death is unpredictable and can happen at any time and as such Muslims should always be prepared for the inevitable and for what is about to occur. “When their time comes they cannot delay it for a single hour nor can they bring it forward by a single hour” [34] (16 : 61). Death is but a gateway from this short but mortal existence to a life of immortality in the afterlife. The Quran always affirms the unlimited mercy and forgiveness of God, but links future life to performance in the present life, from birth to death [36]. The earth is described as a resting place for the purpose of worshipping God and doing good deeds [34] (2 : 20-21).

In Judaism, there are sharply contrasting views of how to think about death. Very common is the belief articulated well by Rabbi Lord Immanuel Jacobovits, the late chief rabbi of England and founder of the modern study of Jewish medical ethics, who writes that the parallelism in Deuteronomy 30 : 15—“behold, I set before you this day life and good and death and evil”—indicates that death is evil. Without discounting that prevalent view, we would like to round out the picture by examining two sources from the Jewish tradition that endorse the belief that death is not the enemy. Rather, in this view, life is a wonderful gift. It is an opportunity to serve the Creator and, for this reason, we do our best to preserve life. “And G-d saw all that He had made, and behold it was very good” (Genesis 1). In the teachings of Rabbi Meir they found written: “And behold it was very good”—behold how good is death.” (*Midrash Genesis Rabbah *9 : 5). The beginning of Genesis describes the creation on each of the first five days; at the end of each day, God looks at what He has created and declares that “it was good.” Yet at the end of the sixth day of Creation, the text is slightly but significantly different—God declares that “it was very good.” What is the significance of the extra word, very? Rabbi Meir teaches that there is something additional, not explicitly mentioned in the text, which is also good. Perhaps, surprisingly, he teaches that this “hidden” final good element of creation is death itself. Another source explains, in greater detail, how it is it that the fact of death's existence is actually something positive: “(The angels) said to the Master of the Universe, “It is better for you to give the Torah to the heavens, since we are holy and pure, and the Torah is holy and pure, and we live (eternally), and the Torah is the tree of life, it's better that it should be given to us!” He said to them, “It cannot be fulfilled in the heavens, as it is written, “It is not found in the land of [eternal] life” (Job 28). Rabbi Nehemia said in the name of Rabbi Yehuda, “Draw a parable to a man who had a son who lacked one finger, and he took him to learn the arts. There was one art which requires all the fingers. After some time his father came to see him and found that he had not learned that art. He asked his teacher, “Why have not you taught him this art?!” He replied, “This art requires all the fingers. Your son is lacking one finger, and you are asking for him to learn this art?” So too God said to the angels, “You cannot fulfill the Torah, for you do not reproduce, you do not have impurity or death or sickness, rather you are all holy!” (*Midrash Psalms 8*). Let us closely examine this text. The Torah is the collective body of God's teachings in general, often used to mean the Five Books of Moses in particular. This text teaches that man is able to fulfill God's commands better than the angels, immortal and perfectly pure, ever could. The very fact of our mortality and our experience with impurity enhances mankind's ability to serve God. In Judaism, impurity is generally a function of contact with the world of the souls (contact with a dead body, giving birth, the unfertilized life ending in menstruation) or, in effect, of contact with death. The angels, who live forever, are actually in this sense lesser beings than mankind, who have contact with death, because one grows as a person as a result of living with the reality of death. This takes shape in numerous different ways. The reality of death provides us with the fundamental attitude of gratitude for our very lives. The fact that our time might run out at any moment provides us with motivation to live our lives well, every single day. Many of our patients report how the experience of illness has taught them valuable lessons, such as self-respect and the willingness to admit that we are not in complete control, and helped them to grow as a person. The same can hold true for friends or family members of someone who is ill. In this view, the creation of death as a part of our world is, in fact, “very good.”

## 4. Challenges in Accepting Palliative Care

Cultural factors can pose difficulties in successfully suggesting palliative care to a patient and his family. Many Muslims believe that palliative care does not preserve life but delays death and postpones one's fate. There are those who will feel discriminated against as a minority or as a result of inferior insurance, or because they believe that decisions were made to free up space for another patient [33]. Palliative care, and especially deescalation of care, is seen as “giving up” or shirking one's duty to heal. Furthermore, immigrant or minority Muslims may feel that inferior care is being given because of their religion or ethnicity or level of insurance, or that the physician is lying to the family, exaggerating a poor prognosis to end care sooner and make way for other patients [33].

In Judaism, some patients or their families might refuse palliative care because they see it as a prohibited form of “giving up” on healing. In particular, they might feel that it demonstrates a less than perfect faith that God will heal. The husband of one of our patients refused palliative care for her because he was worried that doing so would show a deficiency of faith for which his wife would be punished by God. There are, of course, a great variety of approaches in Judaism, and it is never helpful to challenge or “correct” a patient or his/her spouse's view, only to learn what their approach is and work within it.

We would like to present one perspective within the Jewish tradition that may be helpful to those looking to find a Jewish viewpoint more in keeping with the palliative approach. The son of one of our patients asked us, “Do you believe in the Maimonidean or the Nachmanidean approach to doing our part to bring about a miracle?” What he was asking, by referring to the positions of two illustrious rabbis of the Middle Ages, was whether or not it was acceptable for them to take a palliative approach in their father's care while maintaining hope for his miraculous healing, or whether they needed to pursue curative oncology treatments as a way of partnering with God in bringing about the miraculous healing. The debate, in philosophical terms, is whether God simply does the miracles or whether we first need to do our part, futile though we know our efforts will be in bringing about the miracle without God's help, and then God will meet us halfway. But the question, in human, pastoral terms, asks whether we must “do everything” or is it permissible, from the perspective of proper faith, to maintain hope for healing while at the same time focusing on palliation.

An important Jewish source for this discussion is the commentary of Rabbi Shmuel ben Meir on the Babylonian Talmud. The Talmud states, “Rav Amram said in the name of Rav, From three sins a man has no escape every day: from sinful (lustful) thoughts,* iyyun tefilla*, and harmful speech” (Babylonian Talmud, Bava Batra 164b). What could be the meaning of the sin of *iyyun tefila*, literally “close examination of prayer,” that people violate every day? Rabbi Shmuel ben Meir explains as follows: “Some explain that after he prays he thinks in his heart that God will reward him and do what he needs and fulfill his request because he prayed with proper intent (*kavana*).” In other words, one must not be so arrogant as to think that God is somehow required to provide what one asks for. Even if we behave perfectly, or pray perfectly, God does not have to do what we say. We can extend this from prayer to faith. Maintaining perfect faith that He will heal will not necessarily lead God to heal our beloved. And not having perfect faith in their healing will not bring about someone's death. We must preserve perfect faith that God can heal, but we need not act as if we are 100% confident that God will heal. Rather, we must do what is best for the moment, given a realistic understanding of the situation, and maintain hope that things will change for the better. Otherwise, we could end up causing a lot more pain and suffering to the patient, our beloved family member, and that is not what Judaism requires.

To return to the philosophical question of whether or not there is a need to “do everything” in order to partner with God in bringing about a miracle, we would suggest that undergoing all the treatment options recommended by the medical team is already a sufficient means of partnership and that, when palliative care is indicated, it is fine to focus on treating the pain and suffering.

## 5. Avoiding Unnecessary Treatments That Increase or Extend Suffering

Another principle of palliative care is to avoid treatments that add to the amount of suffering without a medical expectation of curing the condition or improving quality of life. What do Islam and Judaism have to say?

When death approaches and is unavoidable, Islam directs that the patient be allowed to die without heroic measures or supreme efforts [37]. Medications and medical technology should be used to enhance the patient's quality of life during his life. At the same time, Islam forbids acts that expedite death. Withdrawing care is permissible in two circumstances in Islam. The first is when a diagnosis of brain death has been made. The second is when the current treatment, be it curative or palliative, is no longer curing or palliating suffering but merely prolonging a natural and inevitable death [33]. The Prophet is quoted as saying “None of you should wish for death because of a calamity befalling him; but if he has to wish for death he should say: O Allah! Keep me alive as long as life is better for me and let me die if death is better for me.”

In Judaism, the belief cited earlier, that death is evil, dovetails with many Jews' desire to “do everything” to try for a cure. Even if the goal is not a cure, one common approach is to do whatever is possible to extend life, since one moment of life in this world is more valuable than all the world to come. In contrast to those views, we will bring two recent voices from among the many great Jewish thinkers. Rabbi Moshe Feinstein, a leading American Jewish legal thinker of the 20th century, in response to a question regarding care of a patient for whom no cure is possible, wrote: “If physicians have no means of healing such a patient or of reducing his suffering, but do know a treatment to keep him alive for a limited time at the current level of suffering, then they should not give him this treatment …. Even great medical experts, if they do not know how to heal the patient, then they should not give treatments that do not cure, relieve suffering, or give him the strength to endure his suffering, but if it will calm the patient's soul by giving him something, then it must be given” [38] (2 : 74.1). Rabbi Feinstein grounds his ruling in the Talmudic story (Ketubot 104a) of the death of Rabbi Yehuda the Prince, the leader of the Jewish people. Rabbi Yehuda the Prince was very ill and in a lot of pain and the prayers of all his students and the greatest leaders of the age were only effective enough to preserve his life, but not to reduce his suffering. When his handmaid saw how much pain he was in every time he went to the bathroom, she went up to the roof of the building where the rabbis were praying and threw down a jar. When it shattered, they were momentarily distracted from their prayers and Rabbi Yehuda passed away. The handmaid's action is praised—this was a situation where nothing more could be done for him, neither to cure him of illness nor to relieve his suffering, and she stopped the treatment from being given.

Rabbi Shlomo Zalman Auerbach, a leading Israeli Jewish legal thinker of the 20th century, in discussing the case of a dying patient at the end of his life, wrote: “Some are of the opinion that just as the Sabbath must be violated to preserve even temporary life, so it is similarly obligatory to force the patient [to be treated], because he does not own himself so that he [has the right to] relinquish even a moment of life. However, it is reasonable that if the patient is suffering a lot of physical pain, or even if he is in great psychological pain, (although) I believe it is mandatory give him food and oxygen for breathing even against his will, it is permissible to withhold treatment that causes suffering if he requests it. However, if the patient is God fearing and is not mentally confused, it is extremely desirable to explain to him that a single hour of repentance in this world is more valuable than all of the world-to-come, as (it is written) that it is a “privilege” to suffer seven years rather than to die immediately [39] (1 : 91.24)”.

Thus, we see that very significant rabbinic writers do not automatically endorse continuing with treatments, and either indicate that it would be better not to give the treatment unless the patient specifically requests it or at least conclude that it is up to the patient to decide (see Bleich's alternative application of these rulings [40] and Brody's rebuttal [41]). At the same time, elsewhere, they rule that treatments to reduce pain and suffering must be obtained wherever possible [42]. What conceptual analysis underlies their rulings? For Rabbi Feinstein, at least, it seems that the duty to seek and provide healing only applies to treatments that actually help, either by potentially leading to a cure or by improving the patient's condition [25]. If pain and suffering cannot be reduced, and attempts to cure have failed, then these treatments are not in fact helpful. However, if the patient actively wants the treatment, or if it helps him psychologically or spiritually, then an otherwise unhelpful treatment turns into a beneficial one, and as such it should be pursued.

In the cases generally addressed by palliative care, we can consider a second approach, even though it would not have pertained to the cases that Rabbis Feinstein and Auerbach were discussing. They ruled regarding a situation where additional treatment could extend life slightly, but could not reduce suffering. Even in such a case, they said that Jewish ethics did not mandate such treatment. In palliative care cases, however, palliative care can actually reduce suffering. Furthermore, it is important to note that we have no medical reason to expect that continuing treatments will actually extend life as opposed to a purely palliative approach. Treatments given against doctors' advice in the hope of extending life just as often lead to complications that shorten life. In addition, pain and suffering themselves can shorten life (as noted by Jewish thinkers, as well. See Rabbi Waldenberg citing the statement in the Babylonian Talmud, Tractate Ketuvot 62b, “A groan breaks half a person's body” [43]). In one recent study among several showing this same result, patients with nonsmall-cell lung cancer who received early palliative care lived longer than patients receiving standard care, even though fewer patients in the palliative care group received aggressive end-of-life care [44]. Not all studies have shown such a benefit from palliative care, but many have, so at best the question of which approach extends life more is, at this point, a factual tossup. In addition to a longer life, the palliative care group also enjoyed more productive time at a higher quality of life. Thus, in applying the “extending life” approach, we cannot be sure which approach is better, and the decision can then be left to the patient (Jotkowitz and Zivotovsky [25] cite the opinion of Rabbi Waldenberg, mandating treatment in the same case discussed by Rabbis Feinstein and Auerbach above. However, their position is predicated on the assumption that we are discussing a case where additional treatment will extend life. In our cases, where that assumption may well not be valid, he might not disagree).

In addition to these two conceptual approaches, we would like to suggest and endorse a third possibility [45]. In Judaism, Jews have an obligation to make the most of their lives in terms of serving God, fulfilling the commandments, and growing religiously and spiritually. We have to maximize our ability to achieve in these areas, and not only maximize the amount of time that we live. Once we refocus the conversation on the religious goals of life, we realize that palliative care can sometimes be more conducive to fulfilling these tasks than continued treatments that involve difficult side effects. For example, the palliative approach can leave the patient with more strength for doing good deeds, while the aggressive approach might mean that all one's energy is devoted just to getting through the treatment. As Rabbi Auerbach noted, the conversation with patients should revolve around how best to fulfill one's religious duties during the life that remains. With that guiding principle, the decision regarding undergoing aggressive treatment can be left to the patient because he is often best placed to know for himself which approach will best enable him to serve God.

## 6. Psychological and Spiritual Care

Palliative care also mandates caring for the psychological and spiritual needs of the patient. As we have noted, in Judaism, the command to heal implied in the verse, “and you shall return it to him,” means that whatever is in the palliative care team's power to help restore to the patient, we must try to do so. This can include peace of mind and a sense of hope and meaning. Fear is often one of the overwhelming parts of the experience of the seriously ill patient. Judaism puts treatment of such a patient's fears on the same level as their medical needs. In the Talmud (Shabbat 128b), we read that if a blind woman giving birth on the Sabbath asks that a lamp be lit, in violation of the Sabbath, we light the lamp, in keeping with the well-established principle that we violate the Sabbath in order to save a life. In the Talmud, every teaching comes to teach something we would not have known otherwise. We need to understand what exactly is the case being discussed and what new lesson it is teaching about saving a life on the Sabbath. If the midwife herself needed a lamp for the delivery, then she could light it herself, without the pregnant woman requesting it, since in that case the light is needed to safely deliver the woman's baby. However, we already know that law and so the Talmud would not need to include this statement. Additionally, the innovation of this statement cannot be that the need of the woman giving birth for light is sufficient reason to light it on the Sabbath, since this woman is blind and cannot make use of the light. Where, the Talmud asks, is the new threat to life that motivates the addition of this permissive ruling? The Talmud explains that this will calm her fears that there is not enough light for the others to see her needs and respond to them quickly. Thus, fear alone is considered a life and death condition in the context of medical treatments. The palliative care team can be very helpful in addressing patients' fears, and in this additional way even help to “save a life.” By listening to patients' fears, acknowledging and accepting them, the strength of the fears can be diminished. In addition, by engaging patients in a life review, thinking about the good things that they have already enjoyed in life, the fear for the future can take its place in the larger picture of thinking about one's life in its entirety.

A common spiritual need our patients face is despair, or the lack of hope. Very sick patients might despair not only for their physical health, but they might also despair of their life having any meaning anymore. The palliative care team can help in the process of finding meaning even at this stage. As we noted in our examination of the *Midrash* from Psalms, the encounter with death can actually help facilitate spiritual growth. This can be a time for even greater connectedness with God. It is also worth remembering and reminding patients that even at this point in life, religious good deeds (*mitzvot)* can still be done, and every second of faith or prayer or good deeds is invaluable.

Spiritual care is a vital part of care for the Muslim patient. The spiritual aspect of the Muslim patient is very important in preserving calmness and general wellbeing: disruption to the balance causes illness or worsens existing illness. Abu-Bakr Al-Razi was among the first to present the subject in his book “Spiritual Medicine” (*Al-Tib Al-Ruhani*) [46]. The reading of special verses from the Quran constitutes the cornerstone of spiritual healing. The first to present the subject was Abu-Zaid Al-Balkhi (b. 850, d. 934), who wrote the book “Sustenance for Body and Soul” (*Masalih al-Abdan wa al-Anfus*), in which he stressed the importance of the combined treatment of body and soul. He criticized the doctors who, in his opinion, were interested only in findings about the body when treating illnesses and neglected the emotional and spiritual aspects of the patient. Al-Balkhi stressed the importance of treatment by means of looking at beautiful pictures (guided imagery) and listening to beautiful music (music therapy). Muslims find the greatest solace and comfort in the remembrance of God. During illness, the Muslim patient should set for himself these spiritual goals.

1. Muslims are expected to seek God's help with patience and prayer, increase the remembrance of God to obtain peace, ask for forgiveness, give more in charity, and read or listen to more of the Quran.
2. Muslims repeat the saying “To God we belong and to Him is our return” to ease the shock of death.
3. Atonement (*Tauba*): this is done by experiencing a genuine sense of remorse for one's transgressions and a removal of the unhealthy effects of that state by turning to God and seeking divine grace through prayer, charity, and a sincere resolution not to return to the destructive patterns of the past.

Patients need to make peace with God through religious duties in order to meet God free of sins, and also to make peace with relatives and friends. When a Muslim individual is dying, several things may be comforting to the patient and the family: (a) turning the patient on his/her right side to face Mecca; (b) letting those visiting the patient recite the prayer of allegiance to Allah, and encouraging the dying person to recite it also, if possible. If the patient is unable, another Muslim should recite it; (c) having friends and loved ones pray that mercy, forgiveness, and the blessing of Allah be given to the deceased; (d) reading specific verses from the Quran; (e) helping the dying person overcome the fear of death [47].

## 7. Conclusion—Doctors' Duties

The medical team should keep a number of things in mind in working with a Muslim patient. Health care should inform the patient of diagnosis and prognosis, but should not give a specific estimated life expectancy at any point, since life is in the hands of God, not in the physicians' hands. The patient needs to make peace with God through religious duties, so as to meet God free of sin, and with relatives and friends. This will enable the patient to finish the “unfinished business.”

Truth telling: telling lies is considered a great sin according to the Islamic faith. The Prophet (peace be upon him) said “the signs of a hypocrite are three: whenever he speaks, he tells a lie; whenever he promises, he breaks it; and if you trust him, he proves to be dishonest” [31]. In addition, doctors need to be sensitive to patients' fears that the suggestion of palliative care is a form of discrimination, pushing the patient aside to make room for other patients.

Health care professionals should adopt cultural competence and sensible awareness when caring for Muslim patients and family. A holistic approach to health care demands staff understanding of Islamic belief, religious practice, spiritual beliefs, cultural mores, and social background. With the open borders strategy and population shift from East to West, it is crucial that physicians and nurses be trans-cultural with sensitivity to spiritual needs of their patients. The spiritual treatment of the Muslim patient in general and the terminally ill patient in particular is essential in easing the patient's pain and suffering. The patient must be listened to and the differences in his values and faith must be accepted, reflecting sensitivity and mutual respect and avoiding judging and engaging in prejudice. Improving communication and mutual respect is the basis of achieving the best medical treatment with conflict and stress reduction with the patient and family, and a more satisfied and rewarding practice for the caregiver. Spiritual history and assessment are vital to implementing holistic care, preventing confrontations and embarrassment, and finally ensuring a better quality of life of the acute or terminally ill Muslim patient and family.

In working with the Jewish patient, one must always remember the very diverse set of beliefs and practices to be found among Jews and listen closely to understand the set of beliefs out of which a particular patient or family is operating. Palliative care itself, whether or not it is pursued along with standard care, and regardless of how one thinks of its philosophical underpinnings, is mandated in much if not all of Judaism and should certainly be brought up in conversations with the patient or his/her family in an appropriately sensitive manner. Psychological and spiritual care are a crucial part of the care that needs to be provided, in consonance with the patient's needs and wishes, which may be more or less “religious.”
